# BDNF, GDNF, NGF and Klotho levels and neurocognitive functions in acute term of schizophrenia

**DOI:** 10.1186/s12888-021-03578-4

**Published:** 2021-11-11

**Authors:** Betul Aslan Turkmen, Esra Yazici, Derya Guzel Erdogan, Mehmet Akif Suda, Ahmet Bulent Yazici

**Affiliations:** 1 Department of Psychiatry, Sakarya Training and Research Hospital, Sakarya, Turkey; 2 Department of Psychiatry, Sakarya University, Medical Faculty, Sakarya, Turkey; 3 Department of Physiology, Sakarya University, Medical Faculty, Sakarya, Turkey

**Keywords:** Klotho, Neurodegeneration, Cognitive functions, Neurotrophic factors, Schizophrenia

## Abstract

**Background:**

Klotho and its relationship with neurotrophic factors and cognition in schizophrenia has not yet been investigated. In this study, the hypothesis that the blood serum levels of BDNF, GDNF, NGF and Klotho in schizophrenia patients and healthy controls would be related to cognitive functions was investigated.

**Methods:**

In this study, two groups were assessed: schizophrenia patients (case group) who were hospitalised in the Psychiatry Clinic of Sakarya University Training and Research Hospital and healthy volunteers (control group). The patients were evaluated on the 1st and 20th days of their hospitalisation with the Positive and Negative Syndrome Scale (PANSS), the Brief Psychiatric Rating Scale (BPRS), the General Assessment of Functioning Scale (GAF) and the Clinical Global Impression Scale (CGI). For cognitive assessment, both groups were evaluated with the Wechsler Memory Scale-Visual Production Subtest (Wechsler Memory Scale III-Visual Reproduction Subtest) and the Stroop test.

**Results:**

BDNF, GDNF, NGF and Klotho levels were lower in schizophrenia patients than in healthy controls. In the schizophrenia patients, on the 20th day of treatment, there was a statistically significant increase in BDNF compared to the 1st day of treatment. BDNF, GDNF and Klotho showed positive correlations with some cognitive functions in the healthy controls. BDNF, GDNF, NGF and Klotho levels were intercorrelated and predictive of each other in both groups.

**Conclusion:**

This study suggests a relationship between cognitive functions, neurotrophic factors and Klotho. Most of the results are the first of their kind in the extant literature, while other results are either similar to or divergent from those generated in previous studies. Therefore, new, enhanced studies are needed to clarify the role of Klotho and neurotrophic factors in schizophrenia.

**Supplementary Information:**

The online version contains supplementary material available at 10.1186/s12888-021-03578-4.

## Introduction

Schizophrenia is a chronic, progressive disorder with both neurodevelopmental and neurodegenerative features and periods of remission and recurrence. The general prevalence of schizophrenia in society has been determined to be approximately 1%; goes with severe destruction and leads to disability. The main symptoms of the disease are both positive (e.g. delusions, hallucinations) and negative (e.g. blunted affect, social withdrawal, depression) and include the loss of cognitive functions (e.g. memory and executive functions) [1–3].

Many hypotheses have been proposed regarding the emergence of schizophrenia, one of which pertains to neurodegeneration. There are many factors that lead to neurodegeneration, among the most important of which is a decrease in neuroplasticity. Neuroplasticity is the ability of the nervous system to adapt its structural and functional properties to internal and external stimuli [4].

There are many different mechanisms that provide neuroplasticity in the central nervous system, one of which are changes in the activities of neurotrophic factors [5, 6]. Brain-derived neurotrophic factor (BDNF), glial cell line-derived neurotrophic factor (GDNF) and neuronal growth factor (NGF) are among the most studied neurotrophic factors in schizophrenia. Klotho, on the other hand, is a relatively new marker, for which few studies in schizophrenia patients have been conducted [7].

BDNF is commonly found in the brain and has an important function in memory and learning by increasing synaptic connectivity and neuroplasticity [8]. While serum BDNF levels were found to be low in most studies of schizophrenia [9–14], a few studies have revealed high serum BDNF levels [15, 16].

GDNF and BDNF are markers that play a fundamental role in synaptic plasticity, which is associated with the pathogenesis and treatment of psychiatric disorders. Although no difference was found between serum GDNF levels in schizophrenia patients and healthy controls in one study, GDNF levels were found to be associated with working memory in healthy controls and attention deficit in schizophrenia patients in the same study [17]. Another study found that serum GDNF levels in schizophrenia patients with a movement disorder called tardive dyskinesia (TD), which is thought to be due to long-term antipsychotic drug use, were lower than in normal controls and patients with schizophrenia without TD. This may indicate a neuroprotective effect of GDNF [18]. In sum, the relationship between GDNF levels and the pathogenesis of schizophrenia has not yet been sufficiently investigated, and therefore new studies are needed. Also, the regulation of NGF in schizophrenia, although important, also needs clarification due to inconsistent findings from clinical trials along with several meta-analyses [19].

Klotho was first discovered by Kuro-o and colleagues in 1997 [20]. Klotho-deficient (Klothoa−/−) mice revealed systemic age-related abnormalities, such as walking disorders, emphysema, osteoporosis, arteriosclerosis, hypomyelination, hippocampal neurodegeneration and cognitive deficits [21–23]. The role of Klotho in psychiatric disorders has not yet been adequately studied. There are a limited number of studies in patients with depression, bipolar mania and schizophrenia, and no enhanced study has been performed to compare the blood serum levels of Klotho and neurotrophic factors with cognitive functions [7].

Many cognitive functions, such as learning, memory, attention, executive functions and working memory, are affected in patients with schizophrenia [24]. Relevant data suggest that Klotho may be a new biomarker in the field of neuroplasticity [25]. In the current study, therefore, the relationship between Klotho and cognitive functions was examined. More specifically, the hypothesis that the neurocognitive test performance of patients with schizophrenia would be worse than that of healthy controls and that the blood serum levels of BDNF, GDNF, NGF and Klotho in schizophrenia patients and healthy controls would be correlated with cognitive functions were investigated. The effects of psychiatric healing on neuroplasticity biomarkers were examined by taking blood from schizophrenia patients on the 1st and 20th days of the assessment.

## Material and methods

### Sampling

In this study, two groups were assessed: schizophrenia patients (case group) who were hospitalised in the Psychiatry Clinic of Sakarya University Training and Research Hospital and healthy volunteers (control group).

Patients who voluntarily stopped taking their medicines, experienced an acute exacerbation of their psychotic symptoms, and were already hospitalised due to their clinical needs were included in the study after being re-diagnosed with schizophrenia according to DSM-V diagnostic criteria and after being confirmed to be in the acute exacerbation period. To avoid possible confounding effects of hormones and gender, only men were included in the study. The patients underwent the usual treatment for schizophrenia and measurements were taken on the 1st and 20th days of treatment. The control group consisted of healthy volunteers who were similar to patients in the case group in terms of age, gender and education. Specifically, 41 patients between the age of 18 and 65 and 43 healthy volunteers, i.e. individuals who did not have any systemic and/or neurological disease that would affect literacy, had no psychiatric diagnosis or did not use psychiatric medication, were included in the study. Approval for the study was obtained from the local ethics committee (Ethics committee no: 16214662/050.01.04/216), and written consent was obtained from all participants or, if necessary, from their legal guardians. All methods were performed in accordance with the Declaration of Helsinki.

### Application and scales

The diagnosis of those in the patient group was first made by a DSM-V-oriented clinical interview conducted by an experienced psychiatrist, and a sociodemographic data form was completed. Simultaneous assessment of disease symptoms and cognitive functions was performed on the first day of hospitalisation (or within the first 3 days of hospitalisation if the patient needed to adapt to the tests). For clinical assessment, the Brief Psychiatric Rating Scale (BPRS), the Positive and Negative Symptom Scale (PANSS), the Clinical Global Impression Scale (CGI) and the General Assessment of Functioning Scale (GAF) were used. For cognitive assessment, the Wechsler Memory Scale-Visual Reproduction Subtest (Wechsler Memory Scale III-Visual Reproduction Subtest) and Stroop test were applied. These tests were repeated on patients 20 days after their first administration.

The sociodemographic data form was completed for the control group participants after confirming that they did not have a psychiatric diagnosis by a DSM-V-oriented clinical interview. For cognitive assessment, the Wechsler Memory Scale-Visual Production Subtest (Wechsler Memory Scale III-Visual Reproduction Subtest) and Stroop test were applied.

### Sociodemographic and clinical data form

The sociodemographic and clinical data form was administered to collect sociodemographic data and disease history data from patients and control group participants.

### Positive and negative syndrome scale (PANSS)

This scale evaluates positive and negative symptoms and general psychopathology in schizophrenia or other psychotic disorders and measures the severity of these symptoms. The scale consists of 30 items and includes a 7-point system for evaluating the severity of symptoms [26]. The validity and reliability of the scale in the Turkish context were verified by Kostakoğlu, Batur et al. (1999).

### Clinical global impression scale (CGI)

This scale was developed by Guy (1976) to evaluate the course of all psychiatric disorders in all age groups for clinical research purposes. The CGI has three subsections: severity of disease, improvement and severity of side effects.

### Global assessment of functioning scale (GAF)

This scale is used to rate psychological, social and occupational functioning. Disruptions in functionality due to physical or environmental constraints are not included. Evaluation with the scale is made by the clinician by giving a score between 1 and 100 for the current or past period. The GAF is implemented according to DSM-IV [27].

### Brief psychiatric rating scale (BPRS)

This clinician-administered scale was developed by Overall and Gorham (1962) and consists of 18 items that measure the severity and variation of psychotic and depressive symptoms in schizophrenia and psychotic disorders. Each item is scored from 0 to 6, and the total score is obtained by summing the scores obtained from the items. A score of 15–20 indicates a minor syndrome, whereas 30 and above demonstrates a major syndrome. The BPRS is mostly used in comparative and follow-up studies. The Turkish adaptation was made by Soykan [28].

### Calculation of chlorpromazine-equivalent dose

To evaluate the relationship between antipsychotic dose and neurotrophic factors the antipsychotic doses were recorded. Different kinds of antipsychotics had been used so chlorpromazine-equivalent dose was used for a common unit. The chlorpromazine-equivalent doses was calculated according to studies by [29, 30].

#### Neuropsychiatric tests

##### Wechsler memory scale revised-visual reproduction subtest

This test was developed by Wechsler [31] and is used to evaluate visual learning and memory functions. The highest score one can receive on this test is 14. In the current evaluation, an immediate memory score and a delayed spontaneous recall score were determined. A Turkish validity and reliability study was performed by Karakaş, Kafadar et al. (1996) and was found to be valid and reliable, similar to the original test.

##### Stroop test

The Stroop test, developed by J. R. Stroop (1935), is a cognitive control test consisting of three parts. More precisely, the Stroop test is a neuropsychological frontal region test used to evaluate functional disorders as a result of brain damage. The adaptation study of the Stroop test for Turkish society was carried out by Karakaş [32].

### Collection of biological samples

During the assesment, 5 ml of venous blood was taken into a biochemistry tube (BD Vacutainer SST TM II Advance plastic tubes, Silica Gel, U.K.) from the case group patients on the 1st day and on the 20th day of their hospitalisation, and during the first interview with participants from the control group. After waiting 2 h for the completion of the coagulation process at room temperature, the venous blood was centrifuged at 10000 g (Thermo Scientific, Centrifuge SL16R, UK) for 15 min. Serum samples were collected in Eppendorf tubes and stored at − 80 °C until the day of analysis of the samples.

### Biochemical analysis

In this study, serum GDNF (Bioassay Technology Laboratory, Catalog No: E0122Hu, China), BDNF (Bioassay Technology Laboratory, Catalog No: E1302Hu, China), NGF (Bioassay Technology Laboratory, Catalog No: E2102Hu, China) and Klotho (Bioassay Technology Laboratory, Catalog No: E2781Hu, China) concentrations were evaluated by the enzyme-linked immunosorbent assay (ELISA) method. Biochemical analysis was performed on serum samples taken on the 1st and 20th days of hospitalisation of the patients in the case group and on the serum samples of control group participants taken during their first interview.

### Statistical analysis

The study data were evaluated through the SPSS 17.00 programme. After the descriptive and frequency analysis, the groups were compared. When the groups were evaluated, a Student’s t-test was used to compare the means of the variables that fit the normal distribution of the Kolmogorov–Smirnov test, and a Mann–Whitney U test was used for the variables that did not fit the normal distribution. In the Kolmogrov-Smirnov test, skewness and kurtosis values were accepted as normally distributed if they were between − 1.5 + 1.5 [33]. Dependent variables were evaluated with a paired samples t-test if they showed a normal distribution, and with a Wilcoxon test if they did not. The PANSS, GAF, CGI, Stroop test and Wechsler Memory Scale scores obtained in this study were linear variables, and these were compared between the groups. Additionally, a Pearson correlation analysis was performed on blood serum levels of BDNF, GDNF, NGF and Klotho. To identify which variables had an impact on levels of neurotrophic factors (e.g. Klotho levels) and to determine the degree to which particular independent variables influenced the dependent variables (neurotrophic factor levels), regression analysis was performed. A regression model was determined according to significant results obtained during previous statistical analysis. Categorical variables were evaluated with chi-square analysis. A significance level of *p* < 0.05 was considered acceptable.

## Results

### Sociodemographic features

In this study, the sample was divided into two groups: a ‘Patient Group’, comprising 42 male schizophrenia patients, and a ‘Control Group’, comprising 43 healthy male participants.

The mean age of the Patient Group was 37.47 ± 10.72, and the mean age of the Control Group was 36.27 ± 10.14. There was no statistically significant difference between the two groups in terms of mean age (t = 0.529, *p* = 0.598). The mean years of education in the patient group was 8.21 ± 3.04 years, and the mean years of education in the control group was 10.04 ± 3.47 years. There was thus a significant difference between the two groups in terms of years of education and education level (Z = -2.46, *p* = 0.014) (Table 1).

**Table 1:** Sociodemographic characteristics and smoking habits of the patient and control groups

	Patient group		Control group		p
Sociodemografical data (***n*** = 85)	n	%	n	%	
**Education (years)**					0,006
**0–5**	16	38,1	10	23,4	
**6–8**	15	35,7	7	16,2	
**9>**	11	26,1	26	60,4	
**Marital status**					<0.001
**Married**	11	26,2	31	72,1	
**Single/ Divorced/Widowed**	31	73,8	12	27,9	
**Occupation**					<0.001
**None**	39	92,8	1	2,4	
**Working**	3	7,2	42	97,6	
**Income of the Household (TL)**					<0.001
**0000–2000**	24	57,1	0	0	
**2000–3000**	14	33,3	15	34,9	
**3000–4000**	2	4,8	16	37,2	
**Over 4000**	2	4,8	12	27,9	
**Cigarette**					<0.001
**Smoker**	35	83,3	26	60,4	
**Not smoker**	7	26,7	17	39,6	
**Region of residence**					< 0.005
**Province**	9	21,4	25	58,1	
**District/Village**	33	78,6	18	41,9	
**Crime event**					<0.001
**Yes**	16	38,1	0	0	
**No**	26	61,9	43	100	
Total	42	100,0	43	100,0	

### Evaluation of clinical features and scales

When the clinical history data of the patient group was examined, the mean disease onset age was 21.80 ± 3.98 years, the mean prodromal period was 21.92 ± 28.44 months, the mean time from the prodromal period to hospital admission was 43.90 ± 56.87 months, the mean length of hospitalisation was 156.90 ± 162.88 days, and the mean number of hospitalisations was 4.59 ± 4.10.

The patients were evaluated on the 1st and 20th days of their hospitalisation with the PANSS, the BPRS, the GAF and the CGI, and a significant improvement was detected in all scales on the 20th day. Table 2 presents the comparison of the scale scores of the patients on the 1st and 20th days of hospitalisation.

**Table 2:** Comparison of clinical scale scores on the 1st and 20th days of the patients

Clinical scales (***n*** = 42)	Patient group day 1				Patient group day 20				
	Mean	SD	Min	Max	Mean	SD	Min	Max	p
**PANSS pozitive**	22,80	7,75	11	41	17,26	5,38	8	30	<0.001
**PANSS negative**	23,54	7,50	9	41	18,85	6,66	7	34	<0.001
**PANSS general**	37,90	8,27	19	59	30,39	8,38	18	60	<0.001
**PANSS total**	84,54	18,08	56	138	66,43	15,38	36	95	<0.001
**BPRS**	33,95	11,75	6	65	25,14	8,48	9	45	<0.001
**IGD**	33,5		18	49	44,5		19	80	<0.001
**CGI**									
**Symptom Severity**	4,80	1,13	1	6	3,92	1,05	1	6	<0.001
**Remission**	3,95	0,21	3	4	2,82	0,58	2	4	<0.001
**Adverse Effect**	1,21	0,51	1	3	1,41	0,74	1	3	0.070

Among the classical antipsychotics, haloperidol, zuclopenthixol and chlorpromazine were ordered. Among the atypical antipsychotics, risperidone, paliperidone, olanzapine, quetiapine, aripiprazole, clozapine and amisulpiride were ordered. Six patients received clozapine treatment on the 1st day of hospitalisation, while eight patients received clozapine treatment on the 20th day of hospitalisation. The equivalent dose of chlorpromazine was determined as 557.83 + − 467.67 (0–1725) on the 1st day of hospitalisation and 588.69 + − 409.58 (0–2175) on the 20th day of hospitalisation. Clozapine doses ranged from 25 to 500 mg on Day 1 and 100 to 700 mg on Day 20.

## Evaluation of neuroplasticity markers

### BDNF, GDNF, NGF and Klotho levels in the patient group

**Fig. 1:**
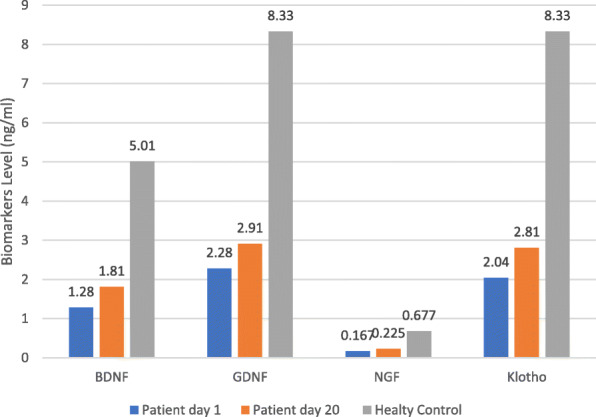
Comparison of groups for the levels of neurotrophic factors and Klotho

The mean BDNF levels of the patients were 1.28 ± 1.93 ng/ml on Day 1 and 1.81 ± 2.91 ng/ml on Day 20. In the paired samples t-test, the BDNF values of the patients on the 20th day were found to be higher than on the 1st day and were statistically significant (*p* < 0.05). In Table 3 and Fig. 1, the comparison of BDNF, GDNF, NGF and Klotho levels in the patient group on the 1st and 20th days is presented.

**Table 3:** Comparison of BDNF, GDNF, NGF, and Klotho levels measured on day one and day 20 of the patients

Neurocognitive biomarker (***n*** = 42)	Patient day 1		Patient day 20		
	Mean	SD	Mean	SD	p
**BDNF (ng/ml)**	1,28	1,93	1,81	2,91	0,032*
**GDNF**	2,28	3,39	2,91	4,16	0,167**
**NGF (pg/ml)**	167,39	260,32	225,51	447,20	0,206*
**KLOTHO (ng/ml)**	2,04	3,14	2,81	2,81	0,096*

*SD* Standard Deviation (* Paired sample T test ** Wilcoxon Test)

### BDNF, GDNF, NGF and Klotho levels of patients and controls on day 1

The BDNF, GDNF, NGF and Klotho levels of the patients on the 1st day were compared with those of the control group participants via an independent samples t-test. The results showed that the BDNF, GDNF, NGF and Klotho levels of the control group participants on the 1st day were higher than those of the patients (*p* < 0.05) (Table 4).

**Table 4:** Comparison of 1st day BDNF, GDNF, NGF, and Klotho Levels of Patient and Control Group

Neurocognitive biomarker (***n*** = 42)	Patient day 1		Control group		
	Ort.	SS	Ort.	SS	P
**BDNF (ng/ml)**	1,28	1,93	5,01	4,60	< 0.001
**GDNF (ng/ml)**	2,28	3,39	8,33	7,13	< 0.001
**NGF (pg/ml)**	167,39	260,32	677,28	732,60	< 0.001
**KLOTHO (ng/ml)**	2,04	3,14	8,33	9,19	< 0,001

#### Correlation with psychiatric symptoms and neurocognitive biomarkers

When the relationship between the clinical scales of the patients on Day 1 and Day 20 and the Klotho, BDNF, GDNF and NGF levels was evaluated, no statistical significance was found (*p* > 0.05).

#### Correlation with antipsychotic doses and neurocognitive biomarkers

There was no correlation between antipsychotic doses (chlorpromazine equivalent) and neurocognitive biomarkers (*p* > 0.05).

## Correlation analysis of neurocognitive tests and BDNF, GDNF, NGF and Klotho levels

### Correlation analysis of BDNF, GDNF, NGF and Klotho levels of patients on day 1 and day 20 and Weschler visual memory test and Stroop test results

No statistical significance was found in the Pearson correlation analyses performed to evaluate the relationship between patients on Day 1 and Day 20 concerning Weschler Visual Memory Test and Stroop test results, and BDNF, GDNF, NGF and Klotho levels (*p* > 0.05).

### Correlation analysis of BDNF, GDNF, NGF and Klotho levels of controls on day 1 and day 20 and Weschler visual memory test and Stroop test results

A negative correlation was found between GDNF levels and Weschler Visual Memory Test results via the Pearson correlation analysis performed to evaluate the relationship between BDNF, GDNF, NGF and Klotho levels and Weschler Visual Memory Test and Stroop test results (*p* < 0.05). As the GDNF level increased in the control group, the 1st and 40th minute test scores decreased (Table 5).

**Table 5:** Correlation analysis of Weschler Visual Memory Test and BDNF GDNF NGF and Klotho levels in the Healthy Control Group

Neurocognitive tests (***n*** = 43 control group)	BDNF	GDNF	NGF	Klotho	
**Weschler Visual Memory Test**					
**Minute 1**	-,238	**-,317**	-,127	-,194	**r**
	,066	**,038**	,418	,212	**p**
**Minute 40**	-,258	**-,304**	-,106	− 181	**r**
	,094	**,047**	,500	,204	**p**

As a result of the Pearson correlation analysis performed to evaluate the relationship between the Stroop test and BDNF, GDNF, NGF and Klotho levels among control group participants, a positive correlation was found between BDNF, GDNF, NGF and Klotho levels and the time taken to read coloured words and say the colour of the coloured words on the Stroop test (*p* < 0.05). As BDNF, GDNF, NGF and Klotho levels increased in the control group, the reading time also increased (Table 6).

**Table 6:** Correlation analysis of Stroop Test and BDNF GDNF NGF and Klotho levels in the healthy control group

Neurocognitive tests (***n*** = 42 control group)	BDNF	GDNF	NGF	Klotho	
**STROOP TEST**					
Time to read words	,309^*^	,309^*^	,401^**^	,407^**^	**r**
	**,043**	**,044**	**,008**	**,007**	**p**
Time to say the word color	,467^**^	,480^**^	,329^*^	,459^**^	**r**
	**,002**	**, 001**	**,031**	**,002**	**p**
Number of telling the color of the boxes (wrong)	,251	,314^*^	,096	,164	**r**
	,105	**,040**	0,539	,293	**p**

*p < 0,05, **p < 0,001

As a result of the Pearson correlation analysis performed to evaluate the relationship between the Stroop test and BDNF, GDNF, NGF and Klotho levels among control group participants, a positive correlation was found between BDNF, GDNF, NGF and Klotho levels and the number of times the colour of coloured words was incorrectly identified on the Stroop test (*p* < 0.05). As GDNF levels increased, the number of false statements also increased (Table 6).

### Intercorrelation of BDNF, GDNF, NGF and Klotho levels

A Pearson correlation analysis of the intercorrelation of BDNF, GDNF, NGF and Klotho levels was performed in both groups separately (*p* < 0.05).

In the correlation analysis of the intercorrelation of BDNF, GDNF, NGF and Klotho levels of patients and control group participants performed on the 1st and 20th days, all biomarkers were found to be intercorrelated (*p* < 0.05 in both groups).

#### Regression analysis

In the regression analysis performed on the 1st day for patients, BDNF and NGF positively predicted and GDNF negatively predicted Klotho levels (aR2 = 0.992); on the 20th day, only NGF predicted Klotho levels (aR2 = 0.960). In the regression analysis performed on control group participants, BDNF and NGF predicted Klotho levels (aR2 = 0.987). Weschler first moment, Weschler 40th moment, Stroop box time, Stroop coloured word reading time, Stroop coloured word time, Stroop false box time and, alternately, BDNF, GDNF, NGF and Klotho levels were used for the regression model (see Supplement – Tables of Correlation and Regression Analysis).

## Discussion

In this study, the relationship between cognitive functions and BDNF, GDNF, NGF and Klotho levels in 41 patients diagnosed with schizophrenia who had acute psychotic exacerbation and in 43 healthy controls was examined.

When the PANSS, CGI, IGD and BPRS scales of the patients on Day 1 and Day 20 were evaluated, the PANSS and BPRS scale scores on Day 20 were found to be significantly lower than the scale scores on Day 1. When the IGD scores of the patients were evaluated, the scale scores on Day 20 were higher than those on Day 1. The functionality levels of the patients were also shown to increase. When the side-effect sub-parameter was examined in the CGI scale, no statistically significant difference was found, but the severity of the disease decreased; an improvement in the sub-parameter scale score was observed. These scores suggest that the patients were in a recovery period and that they were benefiting from the treatments they were receiving. The clinical features of the patients and their differences from features of the control group participants were generally consistent with the typical clinical features and differences of schizophrenia patients known in the literature and demonstrated in previous studies.

### Comparison of serum marker levels of the patient and control groups

When the BDNF, GDNF, NGF and Klotho levels of the patient and control groups were compared, the levels in the control group were significantly higher. When these levels were compared in patients on Day 1 and Day 20, it was determined that they were higher on Day 20, yet only BDNF levels reached statistical significance. This suggests that patients may experience an increase in neuroplasticity biomarkers with treatment, which may in turn slow down the neurodegenerative process in these patients. However, further studies with a larger sample are needed to verify this effect. In this area, BDNF especially has been the most studied biomarker with respect to the neurodevelopmental hypothesis [34].

In this study, no correlation was found between neurotrophic levels, but various antipsychotics were used and the sample was too small to make subgroups based on one kind of antipsychotic; one type of antipsychotic may have a different impact than another so this impact may be a reason for the lack of correlation. In one study that investigated the effects of antipsychotic drugs on serum and plasma BDNF levels, a positive correlation was found between clozapine dose and BDNF levels in schizophrenia patients using clozapine but, similar to the present study, the same correlation was not found with equivalent doses of typical antipsychotics [35]. While most studies on serum BDNF levels in patients with schizophrenia have found low serum BDNF levels [9–13, 36] [14], in a few studies, BDNF levels were found to be high [15, 16]. In one meta-analysis, serum BDNF levels of patients with schizophrenia were found to be lower than those of healthy controls, regardless of drug use [34]. Although the relationship between changes in the brain and serum BDNF levels due to BDNF expression is not fully understood, it is thought that a genetic predisposition towards schizophrenia may affect BDNF levels. In addition, a few studies have been conducted on the effect of chronic neuroinflammation on BDNF levels in patients with schizophrenia [37]. In addition, it should not be overlooked that BDNF levels are affected by lifestyle factors such as drug use, age, physical exercise, diet and stress, which may be significant for schizophrenia patients [38].

In a meta-analysis examining NGF levels in schizophrenia, decreased peripheral serum NGF levels were found to be associated with the psychopathology of schizophrenia (Qin, Wu et al. 2017). In studies conducted in this area, it has been reported that serum NGF levels are decreased in schizophrenia patients compared to healthy controls; NGF levels and NGF receptor levels have also been shown to decrease in cerebrospinal fluid (CSF) [39]. In one study that investigated NGF levels and antipsychotic drug use, it was determined that antipsychotic drug use increased NGF serum levels [40]. In the present study, serum NGF levels were found to be lower in schizophrenia patients compared to the control group participants, and an increase in serum NGF levels was observed on the 20th day of the treatment as compared to the 1st day, in accordance with the literature. But in the present study, the clinical scales of the patients and Klotho, BDNF, GDNF and NGF levels were assessed on the 1st and 20th days. When the relationship between the two was evaluated, no statistical significance was found. This calls into question the effect of these markers on clinical symptoms. The relationship between low serum NGF levels and structural changes in the brain in schizophrenia patients points to a decrease in gray matter volume, especially in the left midcingulate cortex, in addition to low hippocampal volume [41].

In one study that explored GDNF serum levels in different psychiatric disease groups, serum GDNF levels were found to be lower in schizophrenia patients compared to control group participants [42]. Serum GDNF levels of first-episode psychosis patients who did not use medication were followed for 8 weeks and measured in the 2nd, 4th, 6th and 8th weeks; GDNF levels, which were initially found to be lower in schizophrenia patients than in healthy controls, increased with the use of antipsychotic drugs (Xiao, Ye et al. 2016). In a study evaluating GDNF serum levels and clinical scales, only female patients were included. In this study, no significant difference in serum GDNF levels were found in the healthy control group (Skibinska, Kapelski et al. 2017). In the present study, serum GDNF levels were found to be lower in schizophrenia patients than in the healthy controls.

Klotho is a biomarker that has recently been studied in psychiatric disorders, but there is a limited number of studies measuring these serum levels in schizophrenia patients. GDNF is secreted from glial cells, and morphological and functional defects of glial cells may be associated with the pathogenesis of schizophrenia. Finding low NGF serum levels in patients with schizophrenia can be considered as both a result and a treatment goal [43]. The current study evaluated serum levels of biomarkers but it was shown that serum levels, CSF levels and brain mRNA levels and genes, and even different brain part properties of these molecules, may change in schizophrenia and are important to explaining the pathogenesis of disorders [44]. As such, further studies on CSF and an investigation of their relation to brain areas may enhance understanding in this area.

In one study, patients with schizophrenia who were experiencing either acute exacerbation or remission were compared with healthy controls. Klotho levels were found to be higher in schizophrenia, but no statistical significance was found [7]. However, in other studies, Klotho levels were found to be significantly higher in schizophrenia [25]. In the present study, Klotho levels were found to be statistically significantly lower in patients with schizophrenia than in healthy controls, with a statistically insignificant increase on the 20th day of treatment. These results suggest a dynamic relation between schizophrenia and Klotho, but it remains difficult to determine the reason for the changes in the levels of Klotho with the current data. In this study, the standard deviation values of BDNF, GDNF, NGF and Klotho levels were higher than the mean values and demonstrated a wide range. The reason for this result may be due to the differences in the basal metabolic rates of the individuals included in the study. Basal metabolism is affected by many factors, such as age, gender, genetic factors, body mass index, body fat ratio, physical activity, hormonal changes and drugs [45]. Patients and control group participants with different weights and physical activities were included in the study. Antipsychotics from different groups that affect metabolism were used in the patient group, and all of these factors may have caused the standard deviation of BDNF, GDNF, NGF and Klotho to be high.

### Association with cognitive symptoms

All cognitive domains are commonly affected in schizophrenia. In meta-analysis studies conducted in this area, it was determined that attention, executive functions, memory functions, language functions and global cognitive functions were impaired in schizophrenia patients compared to healthy controls [46, 47]. In the current study, the Stroop test and Weschler Visual Memory Test were applied to both the patient and healthy control groups, and correlation analyses were made with serum levels of BDNF, GDNF, NGF and Klotho. While no correlation was found between neurocognitive tests performed on the 1st and 20th days for patients and neuroplasticity biomarkers, a significant correlation was found in the control group. In a previous study, different from the present results, Klotho was found to be higher in patients with schizophrenia, and it was positively correlated with cognitive functions [25]. In one study conducted in this area, animal modelling with BDNF was performed, and it was shown that BDNF is involved in higher-level functions of the brain, such as perception and regulation of emotions, learning, memory and other neurocognitive functions [48]. In a recent meta-analysis, correlations between serum BDNF and neurocognitive functions were evaluated. Studies involving reasoning and problem solving, attention and all neurocognitive phenotypes were included. In this meta-analysis, while no significant difference was found between attention and serum BDNF levels, reasoning and problem solving and all neurocognitive tests were found to be positively correlated with serum BDNF levels [49]. Studies in the literature have found a relationship between schizophrenia and BDNF in terms of cognitive functions, but this relationship was not confirmed in the current study. This may be related to the number of samples or to the fact that patients were evaluated in the acute phase.

In this study, when neuroplasticity biomarkers and neurocognitive tests were compared in the healthy control group, a negative correlation was found between the 1st and 40th minute test scores on the Weschler Visual Memory Test and GDNF levels. In one recent study, GDNF levels were found to be associated with working memory in healthy controls and attention deficit in schizophrenia patients [17]. Working memory is called short or recent memory, and it refers to the entirety of processes in which necessary information is kept in mind and transferred to long-term memory [50]. The Weschler Visual Memory Test also measures learning and memory functions. The increase in GDNF levels in the healthy control group may have adversely affected working memory. An alternative perspective might be compensatory increases in GDNF levels at the receptor level due to resistance.

In this study, when neuroplasticity biomarkers were compared with neurocognitive tests in the healthy control group, a positive correlation was found between all biomarkers measured between the Stroop test’s time to read coloured words and the time needed to say the colour of coloured words. This situation led to the conclusion that the healthy control group responded by allowing more time to think rather than responding impulsively. In the Stroop test, a positive correlation was found between misreading the colour of coloured words and GDNF, and it was thought that this might be due to distracted attention during the information processing process. It seems that GDNF works through different systems in patients with schizophrenia and in healthy controls.

### Correlation and predictiveness between markers

When BDNF, GDNF, NGF and Klotho levels were evaluated in all groups separately, a significant correlation was found between all markers on the 1st and 20th days in the patients and in the control group participants. NGF is secreted endogenously by peripheral cells, such as immune and nerve cells, smooth muscle cells, melanocytes, fibroblasts and Schwann cells [51].. Klotho is predominantly found in the kidneys but also in the brain, lungs, skeletal muscles, bladder, testes and ovaries [21]. When these two molecules, Klotho and NGF, were examined, it was shown that they were produced by many different organs and cell systems in the peripheral system and played a role in many diseases from neuroinflammation to cancer, such as osteoporosis and aging [20, 52]. These molecules, which are affected by many different systems, may negatively affect GDNF and BDNF via different mechanisms. BDNF and GDNF are mostly involved in central nervous system functions and affect each other positively. In light of all of this information and findings, BDNF seems to work in cooperation with all of the molecules studied.

In the regression analysis, when the healthy control group was evaluated, BDNF, GDNF, NGF and Klotho were found to be predictive of each other. This result implies a strong relationship between BDNF, GDNF, NGF and Klotho, but the mechanism and reasons for this relationship could not be explained. Considering the regression analysis of the patient group and the healthy control group, the studied molecules may be working with different mechanisms in each group.

### Limitations and strengths

The current study had some limitations. Cognitive functions, for example, were evaluated using a limited number of tests. For this reason, it was possible to comment only on certain areas in terms of cognitive functions. It is thus important to repeat the study with the results of the tests performed by evaluating neurocognitive functions in different areas. Serum BDNF, GDNF, NGF and Klotho levels are affected by many variables, and special methods are therefore needed to study these neuropeptides, as they are otherwise difficult to use and have a high margin of error. Considering the prevalence of schizophrenia, increasing the sample size in future studies may increase the strength of the results of the current study.

The strengths of the present study are that, when compared to other studies in the literature, a different variety of neurocognitive tests were included, the diagnosis of schizophrenia was confirmed by an experienced psychiatrist according to DSM-V criteria, and a sample that could be considered homogeneous, in which confounding factors such as substance use and physical diseases were largely excluded, was employed. Serum levels of BDNF, GDNF and NGF in schizophrenia patients have been evaluated previously. In the current study, three neurotrophic factors were evaluated together with neurocognitive tests for the first time. Klotho has started to be studied as a new potential biomarker in psychiatric diseases, and it is thus important to examine its relationship with neurocognitive tests. To date, no previous study has evaluated four biomarkers together, and in this sense the present study makes valuable contributions to the literature.

## Conclusion

This study showed that cognitive functions may be associated with neurotrophic factors and Klotho. BDNF, GDNF, NGF and Klotho levels were lower in schizophrenia patients than in healthy controls; and, in schizophrenia, on the 20th day of treatment, there was a statistically significant increase in BDNF compared to the 1st day. In this study, BDNF, GDNF and Klotho showed positive correlations with some cognitive functions in the healthy control group; GDNF was also shown to function differently from other neurotrophic factors. The evaluation of serum levels of Klotho together with cognitive functions in schizophrenia is a fairly new research pursuit, and as such the present study provided valuable data to the extant literature.

It is recommended that similar studies be carried out on neuropsychiatric diseases in which similar neurodegenerative processes may occur. It is also advisable that this study be expanded to include not only serum levels but also further evaluations of receptors and CSF profiles at the molecular level.

## Supplementary Information

**Additional file 1.**

## Data Availability

The data that support the findings of this study are available upon request from the corresponding author. The data are not publicly available because of privacy and/or ethical restrictions.
