# Supplementary material for: BDNF, GDNF, NGF and Klotho levels and neurocognitive functions in acute term of schizophrenia
Source: BMC Psychiatry. 2021 Nov 11;21:562. doi: 10.1186/s12888-021-03578-4 (PMC8588660; doi:10.1186/s12888-021-03578-4)
Supplement: Supplementary file 1 — Additional file 1. [file 12888_2021_3578_MOESM1_ESM.docx]

**TABLES of SUPPLEMENT**

**Table S1. Correlation analysis between Weschler Visual Memory Test and BDNF GDNF NGF and Klotho levels of the Healthy Control group**

| **NeurocognitiveTests**  **(n=42 Healthy Control group)** | **BDNF** | **GDNF** | **NGF** | **Klotho** |  |
| --- | --- | --- | --- | --- | --- |
| **Weschler Visual Memory Test** |  |  |  |  |  |
| **Minute 1** | **-,238** | **-,317^*^** | **-,127** | **-,194** | **r** |
|  | **,066** | **,038** | **,418** | **,212** | **p** |
| **Minute 40** | **-,258** | **-,304^*^** | **-,106** | **-181** | **r** |
|  | **,094** | **,047** | **,500** | **,204** | **p** |

*** p<0,05, **p<0,001**

**Table S2. Correlation analysis between Stroop Test and BDNF GDNF NGF and Klotho levels of the healthy control group**

| **NeurocognitiveTests**  **(n=42 Healthy Control group)** | **BDNF** | **GDNF** | **NGF** | **Klotho** |  |
| --- | --- | --- | --- | --- | --- |
| **STROOP TEST** |  |  |  |  |  |
| **Reading colored words / Right** | **-,290** | **-,233** | **-,107** | **,198** | **r** |
|  | **,059** | **,132** | **,496** | **,204** | **p** |
| **Reading colored words /Wrong** | **,290** | **,232** | **,107** | **,198** | **r** |
|  | **,059** | **,132** | **,496** | **,204** | **p** |
| **Reading colored words /spontaneous correction** | **-,165** | **-,169** | **-,161** | **-,158** | **r** |
|  | **,291** | **,280** | **,304** | **,310** | **p** |
| **Reading colored words/ Time** | **,309^*^** | **,309^*^** | **,401^**^** | **,407^**^** | **r** |
|  | **,043** | **,044** | **,008** | **,007** | **p** |
| **Say the color of the words / Right** | **-,237** | **-,289** | **-,081** | **-,128** | **r** |
|  | **,126** | **,060** | **,606** | **,414** | **p** |
| **Say the color of the words / Wrong** | **,154** | **,228** | **,050** | **,069** | **r** |
|  | **,326** | **,142** | **,751** | **,662** | **p** |
| **Say the color of the words / spontaneous correction** | **,207** | **,197** | **-,004** | **,115** | **r** |
|  | **,183** | **,205** | **,978** | **,464** | **p** |
| **Say the color of the words / Time** | **,467^**^** | **,480^**^** | **,329^*^** | **,459^**^** | **r** |
|  | **,002** | **, 001** | **,031** | **,002** | **p** |
| **Tell the color of the boxes/ Right** | **-,030** | **-,196** | **-,018** | **-,010** | **r** |
|  | **,848** | **,208** | **,911** | **,949** | **p** |
| **Tell the color of the boxes/ wrong** | **,251** | **,314^*^** | **,096** | **,164** | **r** |
|  | **,105** | **,040** | **0,539** | **,293** | **p** |
| **Tell the color of the boxes/ spontaneous correction** | **,146** | **-,085** | **-,065** | **-,081** | **r** |
|  | **,350** | **,587** | **,677** | **,605** | **p** |
| **Tell the color of the boxes/ Time** | **-231** | **,239** | **253** | **,280** | **r** |
|  | **,137** | **,122** | **,102** | **,069** | **p** |

*** p<0,05, **p<0,001**

**Table S3.** **Correlation analysis of BDNF, GDNF, NGF and Klotho levels on the 20th day of the patients**

| **Neuroplasticity Markers (n=41 Patients 20 day group)** | **BDNF** | **Klotho** | **GDNF** | **NGF** |  |
| --- | --- | --- | --- | --- | --- |
| **BDNF** | **1** | **,932**** | **,726**** | **,965**** | **r** |
|  | **-** | **,000** | **,000** | **,000** | **p** |
| **Klotho** | **,932**** | **1** | **,546**** | **,959**** | **r** |
|  | **,000** | **-** | **,000** | **,000** | **p** |
| **GDNF** | **,726**** | **,546**** | **1** | **,532**** | **r** |
|  | **,000** | **,000** | **-** | **,000** | **p** |
| **NGF** | **,965**** | **,959**** | **,532**** | **1** | **r** |
|  | **,000** | **,000** | **,000** | **-** | **p** |

**Table S4.** **Correlation analysis of BDNF, GDNF, NGF and Klotho levels of the Healthy Control Group**

| **Neuroplasticity Markers (n=41 Healthy Control group)** | **BDNF** | **Klotho** | **GDNF** | **NGF** |  |
| --- | --- | --- | --- | --- | --- |
| **BDNF** | **1** | **,932**** | **,726**** | **,965**** | **r** |
|  | **-** | **,000** | **,000** | **,000** | **p** |
| **Klotho** | **,932**** | **1** | **,546**** | **,959**** | **r** |
|  | **,000** | **-** | **,000** | **,000** | **p** |
| **GDNF** | **,726**** | **,546**** | **1** | **,532**** | **r** |
|  | **,000** | **,000** | **-** | **,000** | **p** |
| **NGF** | **,965**** | **,959**** | **,532**** | **1** | **r** |
|  | **,000** | **,000** | **,000** | **-** | **p** |

*** p<0,05, **p<0,001**

**Table S5. Predictors for BDNF levels on day 1 of patients**

| **Patient group 1st day (n=42)** | **BDNF aR^2^=,995** | | |
| --- | --- | --- | --- |
|  | **Beta** | **t** | **p** |
|  |  |  |  |
| **KLOTHO** | **,724** | **14,729** | **,000** |
| **GDNF** | **,355** | **7,448** | **,000** |
| **NGF** | **-,078** | **-2,173** | **,036** |

**Table S6.** **Predictors for GDNF levels on day 1 of patients**

| **Patient group 1st day (n=42)** | **GDNF aR^2^=,997** | | |
| --- | --- | --- | --- |
|  | **Beta** | **t** | **p** |
| **BDNF** | **1,672** | **7,448** | **,000** |
| **KLOTHO** | **-,909** | **-3,889** | **,000** |
| **NGF** | **,219** | **2,956** | **,005** |

**Tablo S7. Predictors for NGF levels on day 1 of patients**

| **Patient group 1st day (n=42)** | **NGF aR^2^=,905** | | |
| --- | --- | --- | --- |
|  | **Beta** | **t** | **p** |
| **BDNF** | **-1,424** | **-2,173** | **,036** |
| **GDNF** | **,853** | **2,956** | **,005** |
| **KLOTHO** | **1,491** | **3,048** | **,004** |

**Table S8 Predictors for Klotho levels on day 1 of patients**

| **Patient group 1st day (n=42)** | **Klotho aR^2^=,992** | | |
| --- | --- | --- | --- |
|  | **Beta** | **t** | **p** |
| **BDNF** | **1,175** | **14,729** | **,000** |
| **GDNF** | **-,313** | **-3,889** | **,000** |
| **NGF** | **,132** | **3,048** | **,004** |

**Table S9: Predictors for BDNF levels at day 20 of patients**

| **Patient Group 20th day (n=41)** | **BDNF aR^2^=,997** | | |
| --- | --- | --- | --- |
|  | **Beta** | **t** | **p** |
|  |  |  |  |
| **GDNF** | **,300** | **20,166** | **,000** |
| **NGF** | **,844** | **19,334** | **,000** |
| **KLOTHO** | **-,041** | **-,919** | **,364** |

**Table S10: Predictors for GDNF levels at day 20 of patients**

| **Patient Group 20th day (n=41)** | **GDNF aR^2^=,970** | | |
| --- | --- | --- | --- |
|  | **Beta** | **t** | **p** |
| **NGF** | **-2,575** | **-13,382** | **,000** |
| **KLOTHO** | **,162** | **1,155** | **,256** |
| **BDNF** | **3,059** | **20,166** | **,000** |

**Table S11. Predictors for BDNF levels in the Healthy Control Group**

| **Healthy Control Group (n=42)** | **BDNF aR^2^=,968** | | |
| --- | --- | --- | --- |
|  | **Beta** | **t** | **p** |
| **GDNF** | **,328** | **2,590** | **,014** |
| **NGF** | **-,345** | **-2,300** | **,028** |
| **KLOTHO** | **,944** | **4,234** | **,000** |
| **Weschler Visual Memory Test 1 minute** | **-,355** | **-1,435** | **,161** |
| **Weschler Visual Memory Test 40 minutes** | **,312** | **1,263** | **,215** |
| **Tell the color of the boxes/ Time** | **-,048** | **-,517** | **,609** |
| **Reading colored words/ Time** | **-,016** | **-,212** | **,833** |
| **Say the color of the words / Time** | **,028** | **,279** | **,782** |
| **Tell the color of the boxes/ wrong** | **,039** | **,692** | **,494** |

**Table S12. Predictors for GDNF levels in the Healthy Control Group**

| **Healthy Control Group (n=42)** | **GDNF aR^2^=,949** | | |
| --- | --- | --- | --- |
|  | **Beta** | **t** | **p** |
| **Weschler 1. Dk** | **,057** | **,178** | **,860** |
| **Weschler 40. Dk** | **-,156** | **-,496** | **,623** |
| **Tell the color of the boxes/ Time** | **,006** | **,049** | **,961** |
| **Say the color of the words / Time** | **-,058** | **-,615** | **,543** |
| **Reading colored words/ Time** | **-,031** | **-,246** | **,807** |
| **Tell the color of the boxes/ wrong** | **,129** | **1,924** | **,063** |
| **NGF** | **-,215** | **-1,084** | **,286** |
| **KLOTHO** | **,609** | **1,845** | **,074** |
| **BDNF** | **,515** | **2,590** | **,014** |

**Table S13. Predictors for NGF levels in the Healthy Control Group**

| **Healthy Control Group (n=42)** | **NGF aR^2^=,962** | | |
| --- | --- | --- | --- |
|  | **Beta** | **t** | **p** |
| **KLOTHO** | **1,483** | **9,866** | **,000** |
| **Weschler Visual Memory Test 1 minute** | **-,167** | **-,610** | **,546** |
| **Weschler Visual Memory Test 40 minutes** | **,141** | **,519** | **,607** |
| **Tell the color of the boxes/ Time** | **,085** | **,850** | **,401** |
| **Reading colored words/ Time** | **,026** | **,319** | **,752** |
| **Say the color of the words / Time** | **-,201** | **-1,979** | **,056** |
| **Tell the color of the boxes/ wrong** | **,071** | **1,196** | **,240** |
| **BDNF** | **-,401** | **-2,300** | **,028** |
| **GDNF** | **-,160** | **-1,084** | **,286** |

**Table S14. Predictors for Klotho levels in the Healthy Control Group**

| **Healthy Control Group (n=42)** | **KLOTHO aR^2^=,987** | | |
| --- | --- | --- | --- |
|  | **Beta** | **t** | **p** |
| **Weschler Visual Memory Test 1 minute** | **,100** | **,629** | **,534** |
| **Weschler Visual Memory Test 40 minutes** | **-,058** | **-,368** | **,715** |
| **Tell the color of the boxes/ Time** | **-,038** | **-,641** | **,526** |
| **Reading colored words/ Time** | **,030** | **,623** | **,537** |
| **Say the color of the words / Time** | **,094** | **1,563** | **,128** |
| **Tell the color of the boxes/ wrong** | **-,070** | **-2,095** | **,044** |
| **BDNF** | **,373** | **4,234** | **,000** |
| **GDNF** | **,154** | **1,845** | **,074** |
| **NGF** | **,504** | **9,866** | **,000** |
